# Successful correction of tibial bone deformity through multiple surgical procedures, liquid nitrogen-pretreated bone tumor autograft, three-dimensional external fixation, and internal fixation in a patient with primary osteosarcoma: a case report

**DOI:** 10.1186/s12893-015-0112-3

**Published:** 2015-12-07

**Authors:** Akihiko Takeuchi, Norio Yamamoto, Toshiharu Shirai, Hideji Nishida, Katsuhiro Hayashi, Koji Watanabe, Shinji Miwa, Hiroyuki Tsuchiya

**Affiliations:** Department of Orthopedic Surgery, Graduate School of Medical Science, Kanazawa University, Kanazawa, Japan; Department of Orthopedic Surgery, Kyoto Prefectural University of Medicine, Kyoto, Japan; Department of Orthopedic Surgery, Kanazawa Seirei Hospital, Kanazawa, Japan; Department of Orthopedic Surgery, Ishikawa Prefectural Central Hospital, Kanazawa, Japan

**Keywords:** Osteosarcoma, Frozen autograft, Deformity correction, Case report

## Abstract

**Background:**

In a previous report, we described a method of reconstruction using tumor-bearing autograft treated by liquid nitrogen for malignant bone tumor. Here we present the first case of bone deformity correction following a tumor-bearing frozen autograft via three-dimensional computerized reconstruction after multiple surgeries.

**Case presentation:**

A 16-year-old female student presented with pain in the left lower leg and was diagnosed with a low-grade central tibial osteosarcoma. Surgical bone reconstruction was performed using a tumor-bearing frozen autograft. Bone union was achieved at 7 months after the first surgical procedure. However, local tumor recurrence and lung metastases occurred 2 years later, at which time a second surgical procedure was performed. Five years later, the patient developed a 19° varus deformity and underwent a third surgical procedure, during which an osteotomy was performed using the Taylor Spatial Frame three-dimensional external fixation technique. A fourth corrective surgical procedure was performed in which internal fixation was achieved with a locking plate. Two years later, and 10 years after the initial diagnosis of tibial osteosarcoma, the bone deformity was completely corrected, and the patient’s limb function was good.

**Conclusion:**

We present the first report in which a bone deformity due to a primary osteosarcoma was corrected using a tumor-bearing frozen autograft, followed by multiple corrective surgical procedures that included osteotomy, three-dimensional external fixation, and internal fixation.

## Background

Biological reconstruction is a useful procedure after malignant bone tumor excision. Although allografts are widely used [1], tissues are difficult to obtain in some Asian countries for religious reasons [2]. Accordingly, methods of recycling tumor-bearing autografts, including irradiation [3], pasteurization [4], and autoclaving [5], have been developed. We initially established the frozen autograft technique in 1999 [6] and have since reported its usefulness [7]. The advantages of frozen autografts include simplicity and the possibility of preserving proteins, including bone morphogenetic protein (BMP) [8].

Herein, we present a case of deformity due to delayed union after frozen autograft reconstruction of a diaphyseal osteosarcoma of the left tibia that was successfully treated by frozen autograft deformity correction with a ringed-type external fixator. The patient and her parents were informed that her data would be submitted for publication and provided consent for this report.

## Case presentation

**Fig. 1:**
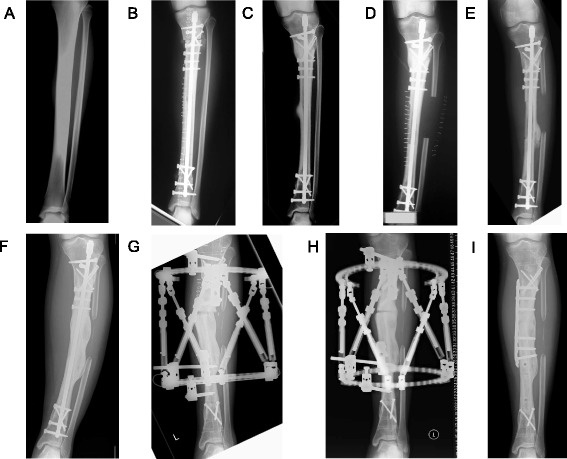
Pre- and postsurgical radiography of a patient with tibial osteosarcoma. **a** Anteroposterior radiograph taken at admission revealed an osteoblastic lesion on the left tibia. **b** Radiograph taken after the first surgery reveals that the frozen autograft was fixed and aligned well. **c** Radiograph taken 17 months after the first surgery shows a recurrent lesion on the surface of the frozen autograft. **d** Radiograph after the second surgery. **e** Radiograph taken 10 months after the second surgery shows a varus deformity with callus formation in the frozen autograft. Bone union with the grafted fibula had not been achieved. **f** Radiograph taken 5 years after the second surgery shows a varus deformity (19°), although union with the grafted fibula had been achieved. **g** Radiograph taken after the third surgery shows the osteotomy site in the frozen autograft and the partial correction of the deformity using the Taylor Spatial Frame. **h** Radiograph taken 9 months after the third surgery shows complete correction of the deformity and partial union of the osteotomy sites. **i** Radiograph taken after the fourth surgery shows fixation of the osteotomy site with a locking plate

**Fig. 2:**
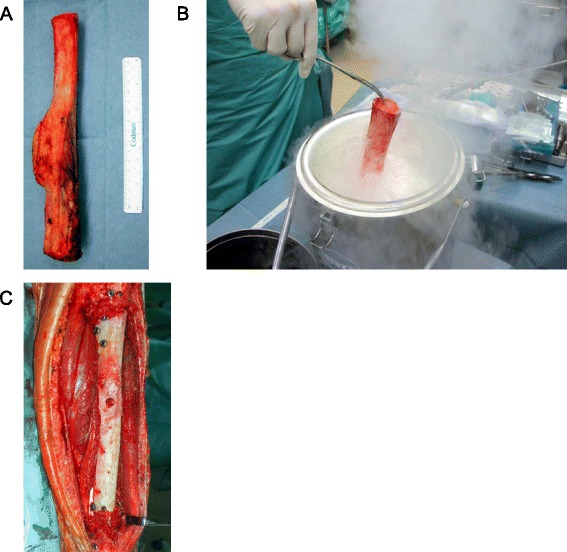
Processing of the resected tumor-bearing bone specimen. **a** Resected specimen measuring 24 cm. **b** The tumor-bearing bone was frozen in a sterilized container of liquid nitrogen for 20 min. **c** After thawing, the frozen autograft was fixed with an intramedullary nail

A 16-year-old Japanese female patient was referred to Kanazawa University Hospital with pain in the left lower leg lasting 2 years. She and her family had no history of malignancy. A plain radiograph of the lower leg revealed an osteoblastic lesion at the diaphysis of the left tibia (Fig. 1a) that was diagnosed as a low-grade central osteosarcoma via open biopsy. No distant metastasis was detected. Wide tumor excision (24 cm) and reconstruction with a tumor-bearing frozen autograft were performed (first surgery; Fig. 1b). Briefly, the tumor-bearing bone was frozen in liquid nitrogen for 20 min (Fig. 2), thawed at room temperature for 15 min, and rinsed in distilled water for 10 min. Bone union was achieved 7 months after surgery.

At 17 months after surgery, local recurrence on the surface of the frozen autograft (Fig. 1c) and lung metastases were detected. A metastasectomy was performed to confirm the histological diagnosis because of the rarity of the distant metastasis of a low-grade central osteosarcoma [9]. The pathological diagnosis was conventional osteosarcoma. Five courses of neoadjuvant chemotherapy were administered, after which tumor excision (hemicortical excision of the frozen autograft) and ipsilateral free fibular grafting were performed (second surgery, 5 months after metastasectomy; Fig. 1d). The local recurrence was histologically confirmed on the surface of the resected frozen autograft. The pathological findings were massive necrotic tumor cells in the osteoid matrix, and the viable tumor cell was eventually detected. Moreover, the viable osteocyte and osteoblasts were detected in the small area of frozen bone. Subsequently, a varus deformity gradually developed, with massive callus formation at the lateral side of the frozen autograft (Fig. 1e).

**Fig. 3:**
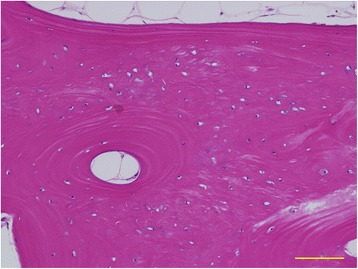
Histological analysis. Large areas of viable osteocytes and osteoblasts were observed. Newly formed trabecular bone was also detected (hematoxylin and eosin staining). The scale bar corresponds to 100 μm

**Fig. 4:**
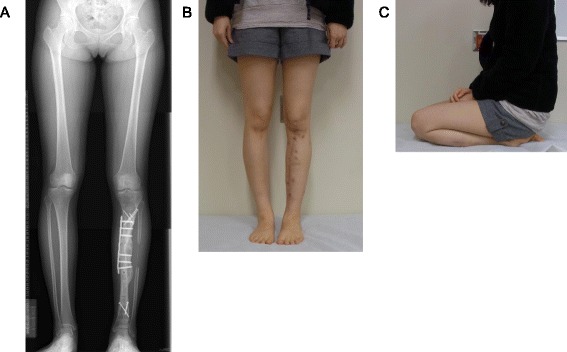
Follow-up radiography and photography. **a** Radiograph taken 2 years after the fourth surgery shows the complete union of the osteotomy site and normal alignment of the left leg. **b** Clinical photo taken from an anteroposterior view demonstrates the normal appearance of the leg. **c** Clinical photo taken from a lateral view shows the normal functioning of the left knee joint

Five years after the second surgery, the varus deformity (19°) was conspicuous despite the achievement of fibular bone union (Fig. 1f). The center of rotation of angulation (CORA) [10] was located in the frozen bone graft; thus, the patient underwent an osteotomy in the frozen bone, application of a Taylor Spatial Frame (TSF) [11], and acute correction (third surgery; Fig. 1g). At the time of osteotomy, a thin slice of the frozen bone that had prevented correction was resected from the osteotomy site. A histological examination revealed the presence of wide areas of viable osteocytes and osteoblasts (Fig. 3). The residual deformity was subjected to gradual correction, and new bone formation was observed in the gap left in the frozen autograft (Fig. 1h). After correction and partial bone union were achieved, TSF was removed, and internal fixation was accomplished using a locking plate (fourth surgery, 9 months after the third surgery; Fig. 1i). Two years after the fourth surgery and 10 years after the initial diagnosis of tibial osteosarcoma, the patient’s deformity was completely corrected, and osteotomy site union was achieved (Fig. 4a). The patient exhibited excellent limb function (Fig. 4b, c).

## Conclusions

The present case demonstrates the osteogenic capacity of a frozen autograft. Although the exact cause of the varus deformity was uncertain, we speculate that mechanical weakness prior to the grafted fibular union caused a minor fracture, thus giving rise to the deformity. To our knowledge, there have been no previous reports of deformity correction or bone unions of fractures in other recycled bone grafts or allografts.

Many autograft recycling techniques have been developed, including extracorporeal radiation [3], pasteurization [4], autoclaving [5], and freezing [6, 7]. The advantages of autograft recycling include good availability, lack of a bone bank requirement, capacity for biological reconstruction, lack of disease transmission, reduced immunological response, soft tissue and ligament attachment capabilities, and availability of a massive bone stock [6]. After we initially developed our tumor-bearing frozen autograft technique in 1999 [6], Igarashi et al. reported the long-term outcomes of these autografts in a study of 36 patients with a mean follow-up of 101 months. They reported graft survival rates of 86.1 % at 5 years and 80.6 % at 10 years [7]. Tanzawa et al. described the histological examination of six frozen autografts removed at a mean of 19.1 months (range: 2–75 months) after implantation because of complications or local tumor recurrence. In all cases, tumor cells had been completely eradicated from the frozen bone, and active osteocytes and osteoblasts were detected in one specimen retrieved 5 months after implantation. Moreover, osteocytes and osteoblasts were observed in broad portions of frozen bones in specimens from three cases retrieved more than 1 year after implantation, indicating the onset of early-stage osteogenesis [12].

The reconstruction of tibial defects following diaphyseal tumor excision is demanding due to the subcutaneous location and poor vascularity [13]. Farfalli et al. reported the clinical and functional outcomes of intercalary segmental allografts [13]. They implanted intercalary tibia segmental allografts in 26 consecutive patients after segmental tumor excisions. Bone union was observed in 24 of the 26 patients during a mean follow-up period of 76 months. Complications were local recurrence in 2 patients, infection in 3, and fracture in 3. Capanna et al. developed a technique that involved combined reconstruction with an allograft shell and vascularized contralateral fibula [14] Ozaki et al. modified this technique using ipsilateral pedicle vascularized fibular graft (VFG) [15]. Li et al. reported 6 patients who underwent tibial intercalary reconstruction using both techniques (2 by Capanna’s technique and 4 by Ozaki’s). Bone union was achieved in 5 of the 6 patients, and the average time was 13.6 months. Complications developed in only 2 patients (1 local recurrence and 1 nonunion) [16]. The combination of a massive allograft and VFG provides immediate mechanical strength, early graft-host junction healing, and lower complication rates, despite the complicated surgical procedures. These techniques have also been applied for recycling sutografts. Mottard et al. performed an irradiated recycling autograft combined with an ipsilateral VFG for 15 patients with diaphyseal tibial tumors. Bone union was achieved in 28 of 30 osteotomy sites, and the mean time was 42.1 (33–55) weeks. Although local complications including wound necrosis, compartment syndrome, nonunion, deformity, infection, and neurological deficit occurred in 7 patients (46 %), limb function was comparable with those who underwent allograft reconstruction [17]. Sugiura et al. reported the clinical outcome of 6 patients who underwent reconstruction using a pasteurized intercalary tibial autograft and VFG composite. Four of the six patients achieved bone union, and the mean period was 10 (7–14) months. Complications were infection in 1 patient, fracture in 1, and pseudarthrosis in 1 patient. The authors described that the patient’s limb functions were satisfactory and compatible to other method [18]. Although we considered the combination of VFG at the initial surgery, we needed a 24-cm excision of the diaphysis. It was difficult to harvest such a large fibular graft for preventing postoperative complications such as the deformity of the ankle and contracture of tendon.

The local recurrence developed on the surface of the frozen autograft in the present case. Yamamoto et al. reported that osteosarcoma cells died after liquid nitrogen treatment both in vitro and in vivo [19]. With regard to the mechanism, Mazur et al. proposed two-factor hypothesis of cryoinjury (intracellular ice versus solute effects) [20]. Therefore, we speculated the local recurrence arose in the soft tissue adjacent to the frozen bone.

In the present case, the osteotomy site was ideal for distraction osteogenesis; however, the osteotomy was performed in the frozen bone autograft out of necessity as CORA [10] was located in this area. Tsuchiya et al. used a rabbit model to analyze frozen autograft bone transport. They demonstrated that bone formation and slow distraction callus maturation occurred after bone transport from frozen devitalized bone. The same authors also presented the case of a 13-year-old girl with a tibial deformity resulting from osteofibrous dysplasia and who was successfully treated with this procedure [21].

Takata et al. assessed the assessed the level and activity of preserved BMPs after treatments at several temperatures, including −196 °C, and found that BMP-7 is significantly better preserved in hypothermic groups. These results suggested that freezing better preserves osteoinductive ability when compared with hyperthermic treatment [8]. These clinical and basic studies supported the ability of frozen autografts to induce osteogenesis, which was not reported for other recycling methods and allografts.

Scarborough et al. reported two cases in which massive allografts were salvaged after fracture by the insertion of additional allografts. However, in these cases, the implantation of additional allografts promoted fracture healing rather than osteogenesis [22]. In addition, Hayashi et al. described the difficult treatment process for nonunion with an autoclaved allograft, despite the implantation of an additional autogenous cancellous bone graft [23]. In contrast, in the present case, the implantation of autogenous cancellous bone contributed to bone union. However, massive callus formation was also observed on the lateral surface of the frozen autograft, which suggested that the frozen autograft harbored potential osteoinductive activity.

In conclusion, a tibial deformity resulting from a tumor-bearing frozen autograft and multiple surgeries was successfully treated via deformity correction with TSF and internal fixation with a locking plate. Although autogenous and cancellous bone grafts were combined in this case, the osteotomy site in the frozen bone completely united with a massive callus formation, indicating the possibility of the osteoinductive activity of the frozen autograft. However, further analysis is necessary to confirm these findings.

## Consent

Written informed consent was obtained from the patient for publication of this Case report and any accompanying images. A copy of the written consent is available for review by the Editor of this journal.

## Abbreviations

BMP: bone morphogenetic protein
CORA: center of rotation of angulation
TSF: taylor spatial frame
VFG: vascularized fibular graft
